# Single‐Cell Atlas of Aging Human Skin Reveals FOSB‐Related Transcriptional Programs and Druggable Targets

**DOI:** 10.1111/jocd.70569

**Published:** 2025-11-26

**Authors:** Youcheng Zhang, Yimin Wang, Dongze Lyu, Yinv Gong, Renpeng Zhou

**Affiliations:** ^1^ Faculty of Synthetic Biology Shenzhen Institute of Advanced Technology, Chinese Academy of Sciences Shenzhen China; ^2^ Department of Plastic and Reconstructive Surgery Shanghai Ninth People's Hospital, Shanghai Jiao Tong University School of Medicine Shanghai China; ^3^ Department of Rheumatology Children's Hospital of Fudan University, National Children's Medical Center Shanghai China

**Keywords:** drug response prediction, FOSB, single‐cell transcriptomic analysis, skin aging

## Abstract

**Background:**

While human skin aging involves complex transcriptional alterations, the cell‐type‐specific regulatory mechanisms and therapeutic targets remain incompletely defined. The study aims to investigate aging‐associated transcriptional programs and drug‐responsive signatures at single‐cell resolution.

**Method:**

Here we performed a single‐cell RNA sequencing of young and elder human skin cells and provided a comprehensive analysis of aging‐related genes, a gene regulatory network with immunohistochemistry (IHC) validation, cell–cell interference as well as potential chemical reactions.

**Result:**

Total 10 distinct cell populations were analyzed based on the scRNA sequencing data. Gene regulatory network analysis showed decreased lactotransferrin gene expression and increased FOSB(+) and DDIT3(+) regulons' regulatory activity. IHC identified the FOSB as an aging‐related transcription factor. Despite uniform MT1 protein distribution across epidermal layers, single‐cell data revealed subcluster‐specific MT1 mRNA dynamics linked to metal ion homeostasis. Drug response prediction experiments revealed possible drug targets and potential antiaging drugs for the skin.

**Conclusion:**

Our data showed the skin cell clusters presented distinct transcriptional signatures during skin aging. We highlighted the versatile expression profiles of metallothionein family genes and identified the aging‐related signature FOSB in the aged skin. We also provided potential target resources for antiaging therapies.

## 1 Introduction

Skin aging is a multifaceted biological process influenced by various factors such as genetic, chronological aging, and environmental exposure [1, 2]. This process results in structural and functional changes in the skin, manifesting as wrinkles, pigmentation, and a decline in the skin and repair capacity [3]. While these phonotypes are well‐documented, their underlying cell‐type‐specific molecular driver, particularly in non‐fibroblast populations, such as keratinocyte subpopulations, remain poorly resolved. For example, the roles of stress‐responsive transcript factor or metal ion homeostasis genes in epidermal aging have not been studied at single‐cell resolution.

Previous studies have revealed age‐related dysregulation of transcription factors such as HES1 in fibroblasts and KLF6 in basal cells during the aging process [4]. Subpopulations were delineated and identified from fibroblasts, where age‐related loss of fibroblast priming was found to have implications for skin aging studies [5]. Despite the established impact of environmental stressors on skin aging, the subpopulation‐specific responses to oxidative stress [6] and metal ion [7] dysregulation—key mediators of environmental damage—have been overlooked. Moreover, the regulatory networks linking stress‐activated transcription factor in aged epidermis remain unexplored.

In this study, we integrated single‐cell transcriptiomics with immunohistochemistry (IHC) validation to analyze aging‐associated changes in keratinocyte. We demonstrated cell‐type‐specific co‐expressed gene modules and gene regulatory modules and the changes in overall cell communication during skin aging. We also mapped the age‐related differentiated gene patterns at the cell type level to the drug molecular signatures to measure the potentiality of the cell‐type‐specific chemical response, facilitating the repurposing and further translations.

## 2 Materials and Methods

### 2.1 Skin Sample Collection

Human skin samples were collected from healthy donors. These samples were categorized into two age groups: Four skin samples in young (14–20 years) groups and four skin samples in elderly (60–76 years) groups.

### 2.2 Cell Isolation, Sequencing, and Library Preparation

Skin samples were collected and immediately stored in ice‐cold phosphate‐buffered saline (PBS, Sigma‐Aldrich). The tissue were minced and enzymatically dissociated in a digestion solution consisting of PBS supplemented with 2 mg/mL collagenase I, 2 mg/mL collagenase IV, 2 mg/mL dispase, and 0.125% trypsin–EDTA. A single‐cell suspension was generated by filtering through a 40‐μm strainer (BD Falcon) and centrifugation at 300 × *g* for 5 min at 4°C. The dissociated cells were sorted by fluorescence‐activated cell sorting (FACS) using a BD Influx instrument. Propidium iodide staining was applied to exclude dead cells. The resulting cells were resuspended for subsequent 10× Genomics sequencing. The library was sequenced on the Illumina Hiseq2500 platform.

### 2.3 Processing scRNA‐Seq Raw Data

Raw sequencing data were processed using cell ranger single‐cell software suite (version 2.2.0, 10×. Genomics). Reads were aligned to the human reference genome (hg19) using the STAR aligner, and gene expression matrices were generated based on unique molecular identifiers (UMIs). Quality control metrics including sequencing depth, gene detection, and mitochondrial gene content were assessed for each sample.

### 2.4 Data Preprocessing and Integration

The Seurat R package (version 5.0) was used for downstream analysis. Cells with high mitochondrial gene content (> 20%) or outlier UMI counts (nFeature_RNA < 200 or > 6000) were excluded. Data normalization and variance stabilization were performed using SCTransform, with mitochondrial gene percentage regressed out. To correct for batch effects, we applied canonical correlation analysis (CCA) integration.

### 2.5 Cell Clustering and Annotation

Principal component analysis (PCA) was conducted on highly variable genes, followed by graph‐based clustering (resolution = 0.5). Cell populations were visualized using Uniform Manifold Approximation and Projection (UMAP). Cell types were annotated based on established markers (e.g., KRT14 for basal keratinocytes, DCN for fibroblasts).

### 2.6 Differential Gene Expression Analysis

Aging‐associated differentially expressed genes (DEGs) were identified for each cell type using the Wilcoxon rank‐sum test (*p* < 0.05, |avg_log_2_FC| > 0.5). Gene Ontology (GO) enrichment analysis was performed on the top 30 DEGs per cell type using the GO_Biological_Process_2023 database.

### 2.7 Gene Regulatory Network Analysis

Gene regulatory networks were inferred using pySCENIC. Briefly, co‐expression modules were generated using GRNBoost2, followed by regulon prediction with cisTarget. Regulon activity was quantified in individual cells using AUCell. Aging‐associated regulons were identified by comparing activity scores between age groups (Mann–Whitney *U* test, *p* < 0.05).

### 2.8 Cell–Cell Communication Analysis

CellPhoneDB (version 3) was used to infer ligand–receptor interactions. Significant interactions *p* < 0.05 were identified by comparing observed interaction scores against a null distribution generated through 1000 permutations of cluster labels.

### 2.9 IHC Staining

Paraffin‐embedded skin sections from young/aged donors were deparaffinized in xylene, rehydrated via graded ethanol, and antigen‐retrieved with citrate buffer (pH 6.0, 95°C, 20 min). Endogenous peroxidase activity was quenched with 3% H_2_O_2_ (10 min, RT), followed by protein blocking with 5% BSA (1 h, RT). Sections were incubated overnight at 4°C with primary antibodies against:MT1 (1:50, #PA5‐121779, Invitrogen); Lactoferrin (1:25, #SL‐53498, Santa Cruz); FOSB (1:25, #2251, CST); JAG1 (1:100, #a12733, Abclonal). After washing, HRP‐conjugated secondary antibodies were incubated for 1 h, followed by hematoxylin staining. Sections were imaged, and ImageJ quantified five fields/sample.

### 2.10 Drug Response Prediction

Drug2Cell was employed to predict cell‐type‐specific responses to known and repurposable compounds. Drug activity scores were calculated based on target gene expression patterns, and significant differences between age groups were assessed using the Mann–Whitney *U* test (*p* < 0.05).

## 3 Result

### 3.1 Identification of Skin Cell Type and Subpopulation Signatures

**FIGURE 1:**
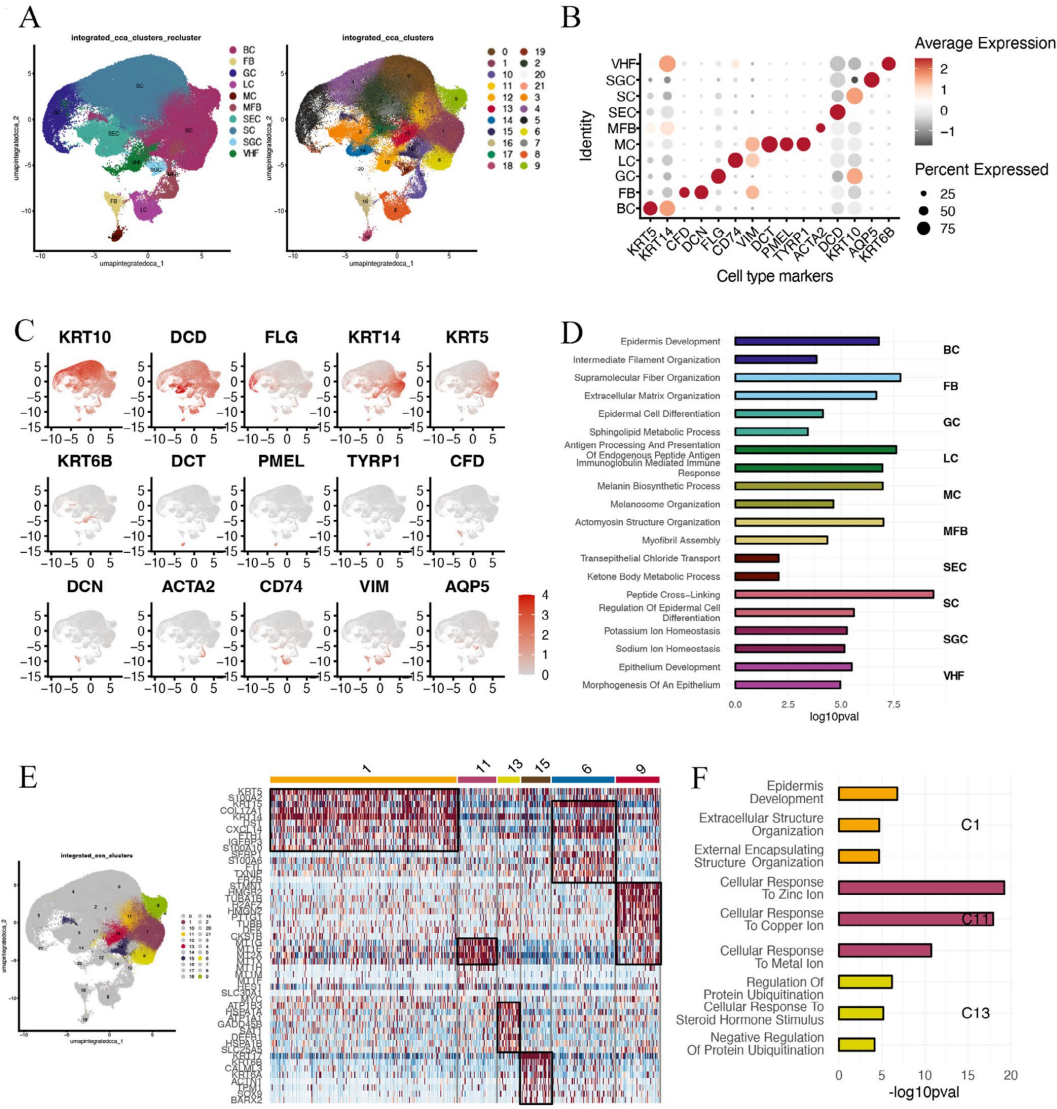
Single‐cell transcriptome profiling revealed skin cell heterogeneity and the subpopulations. (A) Uniform manifold approximation and projection (UMAP) plot showing the cell types of human skin, including (left) board cell population annotation and (right) subpopulations. BC, basal cell; FB, fibroblast; GC, granular cell; LC, langerhans cell; ME, melanocyte; MFB, myofibroblast; SC, spinous cell; SEC, secretory cell; SGC, sweat gland cell; VHF, vellus hair follicle. (B) Dot plot showing the scaled expression of representative cell type markers for each population. (C) UMAP plot showing the expression cell type markers on all skin cells where red color indicates high gene expression levels. (D) Enriched GO biological process terms using the top 30 cluster markers from each cell type. (E) UMAP plot of subpopulations in basal cell (BC) cluster, and scaled gene expression levels in each subcluster where red color indicates high expression levels. (F) Enriched GO terms with top 30 cluster markers in basal cluster C1, C11, C13.

To explore the skin cell heterogeneity, we obtained the human skin from eight healthy individuals of the elder and young group, and conducted single‐cell transcriptome profiling and data analysis. Ten distinct major cell populations were identified by examining the expression patterns of specific cell type markers including basal cell (BC, KRT14, and KRT5), spinous cell (SC, KRT10), granular cell (GC, FLG), secretory cell (SEC, DCD), melanocyte (ME, DCT, PMEL, and TYRP1), fibroblast (FB, CFD and DCN), langerhans cell (LC, CD74, and VIM), sweat gland cell (SGC, AQP5), myofibroblast (MYO, ACTA2) and vellus hair follicle cell (VHF) (Figure 1A,B). The distribution of cell type markers expression patterns was illustrated with the UMAP (Figure 1C). To associate the cell types with biological functions, enrichment analysis for each population with the top 30 marker genes was implemented to reveal the significantly enriched processes relevant to their distinct transcriptional patterns (Figure 1D). Representative biological processes for each cell type were recognized, such as fiber organization and extracellular matrix organization in fibroblast, melanin biosynthetic and melanosome organization in melanocytes, peptide cross‐linking and epidermal cell differentiation in spinous cells. Within the basal cell, six subpopulations (basal cell C1, C6, C9, C11, C13, C15) were delineated (Figure 1E), of which the subclusters exhibited differences on the cluster markers with distinct expression against other clusters. For instance, the largest subpopulation C1 in basal cells identified cluster markers KRT5 and KRT14 with enrichment in epidermis development and extracellular structure Organization, representing the most commonly found basal cell‐specific biological pattern (Figure 1F). Interestingly, we observed that the basal cell C11 cluster showed top differential expression on metallothionein (MT) family genes including MT1G, MT1E, MT1A, and MT1X, which are highly enriched in the cellular response to metal ion processes. Heat shock protein gene family was identified in basal cell C13 such as HSPA1A and HSPA1B as top cluster markers with protein ubiquitination processes enriched (Figure 1F). In the spinous cell, four subpopulations (spinous cell C0, C2, C4, and C7) were highlighted where the S100 calcium‐binding protein gene family was demonstrated as the top responsive markers in C7 and SPINK5 was identified in C4 (Figure S1A). Similarly, three subpopulations were identified in the secretory cell and two in the granular cell (Figure S1B,C). Specifically, in the two subpopulations of the granular cell, late cornified envelope gene family (e.g., LCE2B, LCE1C, LCE6A, and LCE1B) associated with keratinization were identified as cluster markers in C21, indicating the active differentiating transition from C5 granule cell and C21 keratinized granules, and finally, cornified cell (Figure S1C). Two subpopulations were identified in the vellus hair follicle cell (Figure S1D). Notably, while scRNA‐seq revealed subcluster‐specific MT1G/E mRNA dynamics in basal cells (C11), IHC showed uniform MT1 protein distribution across epidermal layers (Figure S2), suggesting posttranscriptional regulation or functional compensation between MT isoforms.

The total number and distribution of cell types captured by single‐cell sequencing were comparable across young and elder donors, indicating that skin cell identity does not alter with age (Figure S3). Therefore, it is necessary to investigate the differential transcriptional changes across different ages.

### 3.2 Transcriptional Variability in Different Cell Types During Human Skin Aging at Single‐Cell Resolution

**FIGURE 2:**
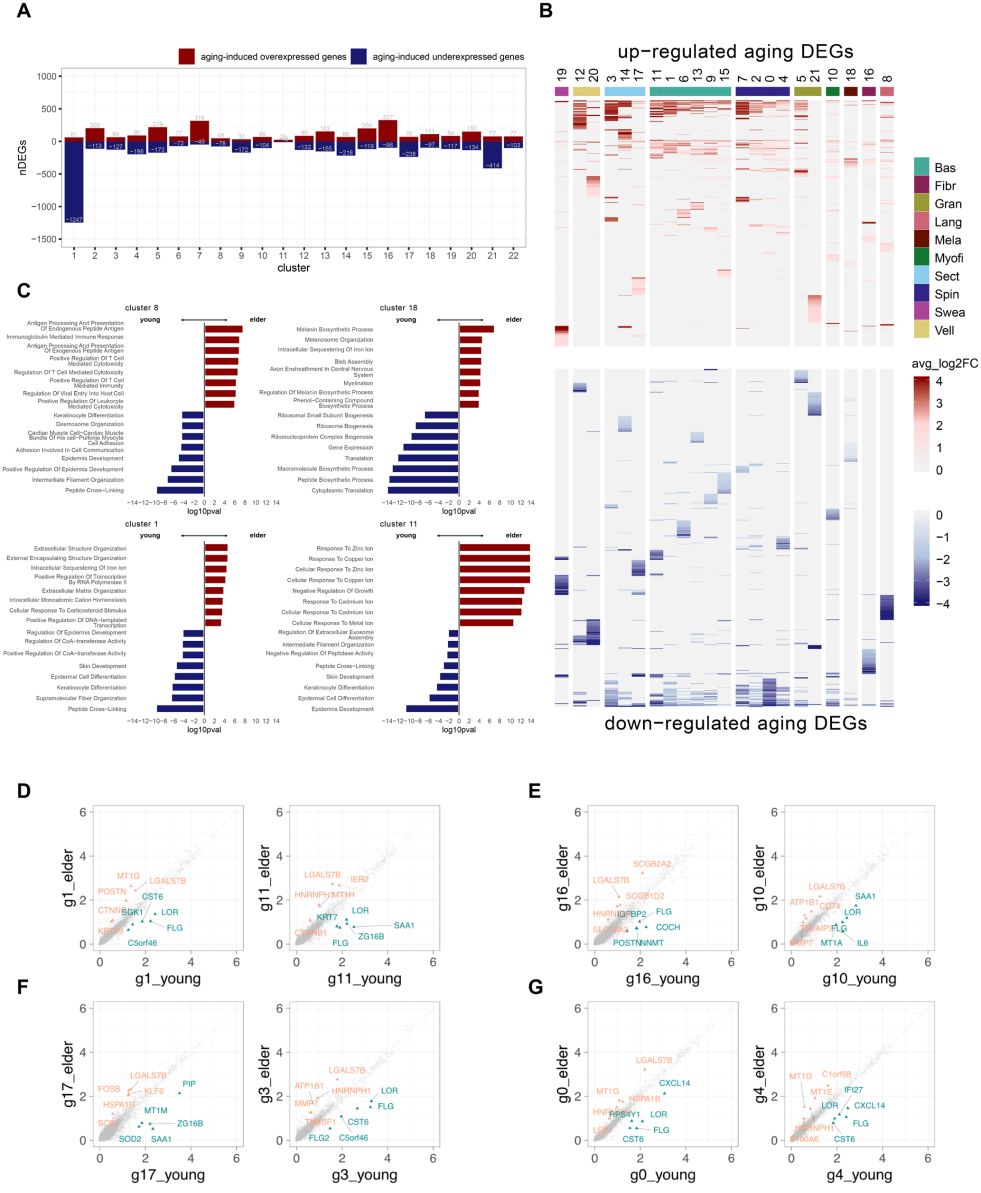
Transcriptional changes of different cell populations during human skin aging. (A) Barplot showing the number of aging‐induced overexpressed DEGs and underexpressed DEGs. (B) Heatmap indicating DEGs with top avg_log_2_FC in each cell population. (C) Mirror bar plot showing the enriched GO terms using the top 30 DEGs from each cell type. (D–G) Scatter plot indicating upregulated (orange) and downregulated DEGs (green) during aging.

Differential analysis was conducted between the elder and young groups within each cell population. The data suggested that aging‐induced unique cell aging molecular profiles change (Figure 2A). It is observed that upregulated genes with top expression change (in darker red) can be majorly found in basal cell (C1, C6, C15), spinous cell (C4), and secretory cell (C14), whereas the top aging downregulated genes were commonly identified across various populations, including spinous cell (C0, C4), vellus follicle cell (C20), immune cell (C8, C16), basal cell (C13), secretory cell (C3) (Figure 2A,B).

We investigated the underlying biological processes associated with these DEGs (Figure 2C). We first examined the differentiated aging processes in melanocyte (C18) and immune cell (C8) clusters as they tend to have specific and well‐described differential patterns. It is shown that the top aging upregulated DEGs of immune cell clusters were highly enriched in antigen processing, immunoglobulin‐mediated immune response as well as immune cells cytotoxicity such as T‐cell and leukocyte.

For melanocytes, the aging DEGs suggested the enrichment of the melanin biosynthetic process and melanosome organization. Similarly, the upregulated DEGs in fibroblast and myofibroblast showed the changed regulation of supramolecular fiber organization (C16) and actomyosin structure organization (C10), meanwhile identifying various negative regulated biological processes such as peptidase activity, endopeptidase activity, and endothelial cell proliferation (Figure S4). Moreover, it is indicated for basal cell populations C1 and C11 that changes in extracellular structure organization and cellular response to metal ions, respectively (Figure 2C). Specifically, the finding in C11 suggested the difference exists in the elder group compared to the young in the regulation of metal toxicity and oxidative stress which is highly involved in zinc and copper. The aging effect also triggers a series of differential processes such as negative regulation of blood coagulation in spinous cell C0, as well as regulation of vascular wound healing in secretory cell C14 (Figure S4).

We highlighted the top DEGs in each cell population between the elder and young groups in Figure 2D–G. For basal cells C1 and C11, galectin 7B (LGALS7B) was significantly expressed higher in the elder skin against the young, which encodes a family of beta‐galactoside‐binding proteins implicated in modulating cell–cell and cell–matrix interactions (Figure 2D). POSTN is involved with a secreted extracellular matrix protein that functions in tissue development and regeneration, and wound healing (Figure 2D,E). We also identified important aging‐related changes regarding the MT family (MT1G, MT1H) which plays a role in the regulation of metal toxicity and oxidative stress, especially involving zinc and copper regulation (Figure 2D). MT was broadly observed to be upregulated in other populations than the basal cell, such as MT1G and MT1E in spinous cells; however, it was downregulated in myofibroblast C10 and secretory cell C17 (MT1A and MT1M) (Figure 2F,G). The above finding demonstrated the versatile expression profiles of MT genes, showing the aging effects on the multifactorial regulation of MT. We also observed widespread and significant downregulation of loricin (LOR) and filaggrin (FLG) (Figure 2D–G), which may explain the barrier dysfunction in aged skin.

### 3.3 Skin Aging‐Related Gene Modules and Regulatory Function Alterations

**FIGURE 3:**
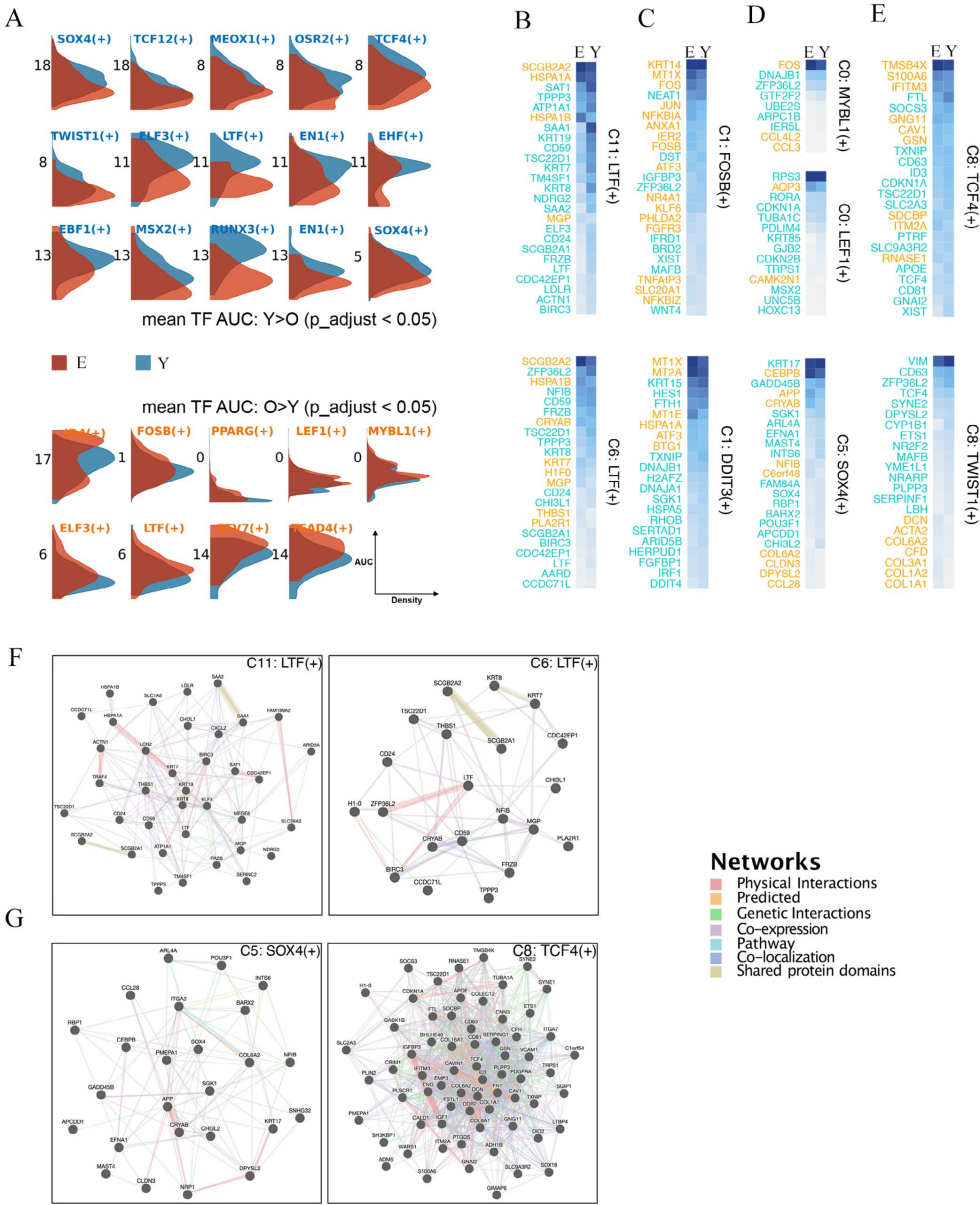
Gene regulatory alteration of different cell populations during human skin aging. (A) Histogram indicating different regulons (transcription factor and the target genes) changes by comparing elder cells (red) and young cells (blue), where the *y*‐axis showed the AUC regulons activity and the *x*‐axis showed the density. Names colored in blue represented the regulons showed decreased activity in aged cells whereas names in red showed the regulons had increased TF activity in aged samples. (B–E) Gene expression changes between the elder group and the young group, in which the *y*‐axis showed a subset of composited genes in the regulons. Full gene sets can be found in Figure S6. (F–G) The gene–gene relationship among the regulon genes with significant differentiated expression.

To explore the regulatory behavior difference associated with skin aging, we predicted the transcription factors (TF) regulons via gene regulatory network analysis on cells from the elder and young groups separately, identified the specific regulons for each cell population with regulon specificity scoring (Figure S5). We identified 11 regulons with significantly increased TF regulon activities in the elder group compared to the young, and 28 regulons with decreased activities (Figure 3A and the full list on Figure S6). Within the basal cell subpopulation, 12 regulons with diverse changes were highlighted due to aging, of which 8 had increased activity while four were decreased (Figure S6).

We compared the expression difference of the corresponding TFs and their target genes in the elder group with those in the young group. Figure 3B–D illustrated the subsets of composed genes in the regulons with top abundance, where genes in orange or green indicated their upregulated or downregulated status in the elder group respectively (full gene list in Figure S7). Although the activity of LTF(+) reacted differently in basal C11 and C6, the expression of the lactotransferrin (LTF) gene was observed to decline in both subpopulations in the elder skin (Figure 3B). IHC showed uniform LTF protein distribution across epidermal layers in both age groups (Figure S8), indicating subcluster‐specific transcriptional regulation uncoupled from bulk protein abundance.

Besides, FOSB(+) and DDIT3(+) regulons are identified with increased regulatory activity in basal C1 (Figure 3C). Consistent with scRNA‐seq data, IHC confirmed elevated FOSB protein expression in aged basal keratinocytes (Figure S9), reinforcing its role as a key aging‐related transcription factor. By further investigating the target genes in FOSB(+) and DDIT3(+) regulons, we noticed that the MTs gene was highly involved, implying that the MT‐regulated metal ion homeostasis could be one of the important factors that influence the cellular process of proliferation and death. Moreover, we identified in spinous cell C0 the increased activity of MYBL1(+) associated with cell cycle regulation with representative target genes such as FOS, DNAJB1, and ZFP36L2, as well as LEF1(+) related to cell state transition and follicle morphogenesis with target genes RPS3, AQP3, RORA, etc. (Figure 3D). In addition, Figure 3F illustrates the different regulon target gene compositions of LTF(+) with significant differentiated expression levels in C11 and C6. It indicated the distinct patterns across cell subclusters of basal cells. For the granular cell C5, decreased activity of SOX4(+) in the elder skin was observed with changed levels of KRT17, CEBPB, GADD45B, APP, etc., which affect the epithelium development and cell migration processes. We also showed the relationship of these differentiated target genes with physical interactions between APP‐CRYAB, NRP1‐DPYSL2‐KRT17, as well as genetic interaction patterns with ARL4A, NF1B, INTS6, and SGK1 (Figure 3G). As for the immune cell C8, overall declined regulatory patterns were noted, where TCF4(+) binds to the immunoglobulin enhancer Mu‐E5/KE5‐motif with IFITM3 interferon‐induced antiviral factor, CAV1 the TLR4‐mediated immune response regulator, CD63 and so on (Figure 3E). The physical interaction across multiple genes such as TCF4, ID3, CD63, CD81, APOE, CDN, and COL family was observed (Figure 3G).

### 3.4 Identification of Skin Aging‐Related Changes in Intercellular Communication

**FIGURE 4:**
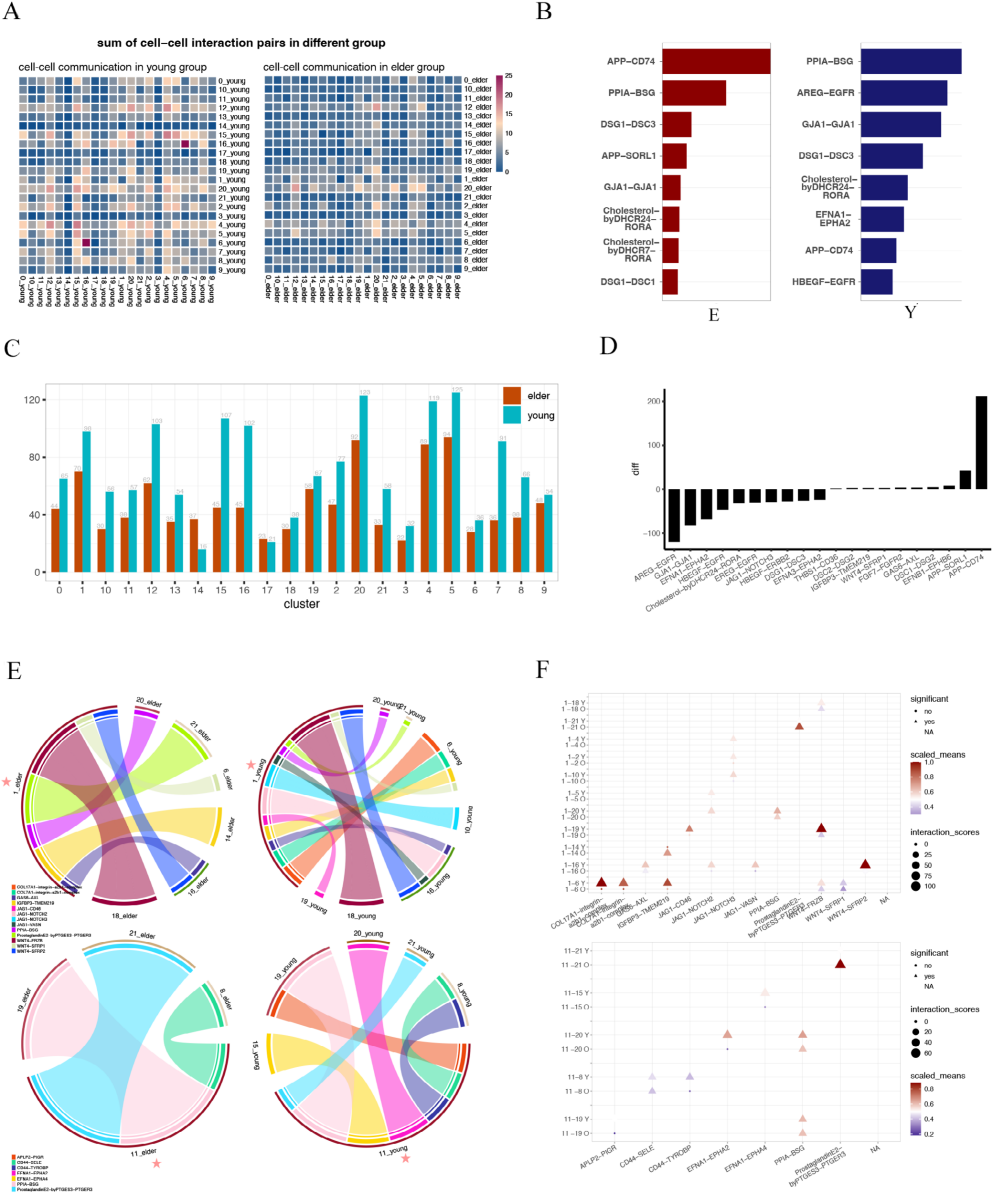
Aging‐induced signaling changes across different cell populations. (A) The overview of cell–cell interaction pairs in the young group (left) and the elder group (right). (B) The top signaling pathways with the most frequency in the elder cell (left) and the young cell (right). (C) The changes in the total interaction pairs in each cell population. (D) The top signaling pathways with the most changes due to skin aging. (E) The chord diagram shows the interaction between each cell cluster using a specific cell cluster as the source cell cluster (ligand) and the other clusters as the target group (receptor), where the star mark indicates the source cluster. (F) The interaction score of signaling corresponds to the left chord diagram.

We conducted the cell–cell communication inference, and data showed that the number of total cell–cell interaction pairs generally declines in the aged skin cells (Figure 4A). We summarized the top signaling with the most frequency, where it showed that APP‐CD74, PPIA‐BSG, and DSG1‐DSC3 are most commonly identified in the elder skin cells (Figure 4B). For the elder skin, signaling associated with immune response (e.g., APP‐CD74) and proliferation (e.g., PPIA‐BSG) was highlighted. The difference in the signaling between the elder and young in each cluster is measured, among which basal C15, immune cell C16, and spinous cell C7 reduced the most number of interactions, while secretory cell C14 and C17 showed an increased interaction (Figure 4C). Furthermore, of all the signaling, AREG‐EGFR, EFNA1‐EPHA2, and JAG1‐NOTCH3 are indicated as the most frequent loss of interaction (Figure 4D).

We further illustrated in Figure 4E,F the significantly altered signaling across different cell populations during the aging process, with a focus on one specific cluster as the source (marked with a red star) while other cell types as a target. It is shown that in Basal C1, the interaction of WNT4 signaling (WNT4−FRZB, WNT4−SFRP1, and WNT4−SFRP2) significantly reduced in the elder group, which is important for preventing aging, wound healing, and inflammation, suggesting that aged skin basal cell could be less active to the relevant processes. Despite transcriptional changes in JAG1 signaling pathways (Figure 4D), IHC analysis did not detect significant age‐related differences in JAG1 protein levels (Figure S10), implying potential noncanonical signaling mechanisms or compensatory regulation. While JAG1 signaling pathways showed transcriptional alterations, the lack of protein‐level changes suggests their contribution to skin aging may be context‐dependent or require alternative ligands/receptors.

### 3.5 Potential Chemical Reactions on Different Cell Clusters Across Aging

In the end, to explore the potential chemical reactions to skin aging, aiming to identify (1) the unknown mechanism and targets of currently approved skin‐aging drugs and (2) the potential new drugs responsive to aged skin cells for future repurposing, we performed drug response prediction experiment with Drug2Cell based on gene group activity evaluation on different cell population [8].

**FIGURE 5:**
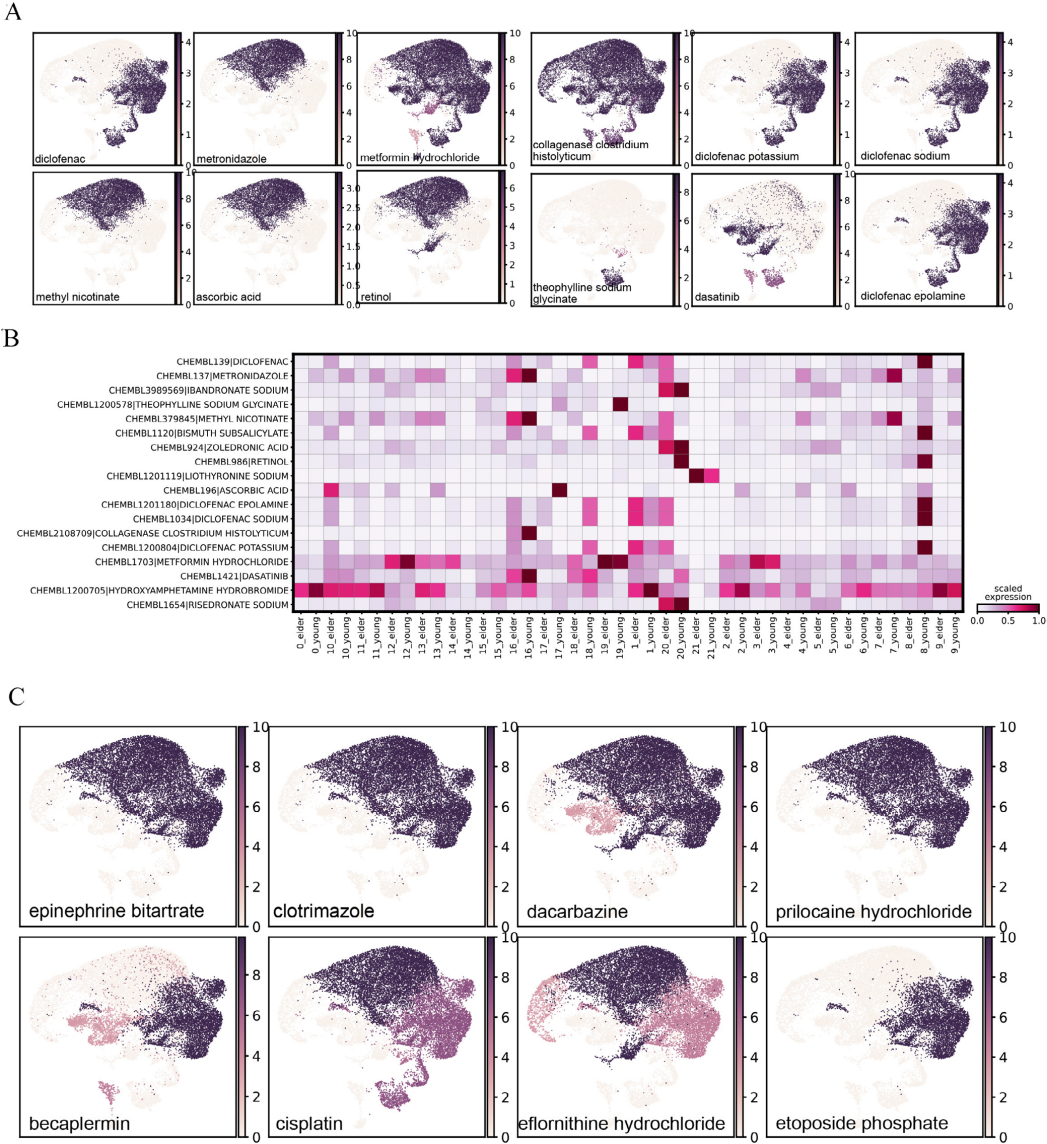
Drug‐cell response (activity) prediction identifying specific cellular targets of compounds with drug properties. (A) UMAP plot indicating the cellular response to a list of known skin‐aging drugs, where dark color measured with logPval suggests the significant cell response changes between the aged cell and the young cell. (B) Feature‐scaled heatmap showing the most responsive cell population that contributes to the overall drug‐cell activity. (C) Drug‐cell activity prediction on a list of drugs with repurposing potential.

To examine the drug responses of known drugs, we verified a list of known skin‐aging drugs that showed a significant change of response between the aged skin and the young skin, including metformin, retinol, collagenase *Clostridium histolyticum* , diclofenac, metronidazole, dasatinib, methyl nicotinate, liothyronine, etc. (Figure 5A). To further evaluate the responsive gene score in the cell subpopulation and highlight the representative cell types that contribute most to the drug, we scaled the gene activity by each drug and found that for metformin, secretory cell C3 and follicle hair cell C12 contribute most to the drug gene activity (Figure 5B). We also compared the gene activity difference between age groups and observed that in most of the cell types cluster, aged cells received a higher drug‐response gene activity on metformin, such as spinous C0, melanocytes C18, immune C8, basal C6, etc., while only spinous C4 and basal C15 showed the reverse trend (Figure 5B). For collagenase *Clostridium histolyticum* , fibroblast C16 gained the highest activity and contributed most to the drug response. Diclofenac is a multi‐target drug with improvement in signs of photodamaged skin and is identified as responsive in the basal cell. Besides, basal C1 and immune C8 respond actively to Diclofenac. Methyl Nicotinate is enriched in multiple cell types such as spinous cells. Several other drugs have unknown mechanisms and targets so far such as metronidazole and retinol.

To discover a potential skin aging–responsive drug for repurposing, we retrieved a new list from CHEMBL with Experimental Factor Ontology (EFO) terms related to skin and repeated the same experimental procedure including Drug2Cell score computing and statistical test between the elder cell and the young. We identified several compounds as candidates possibly reacting to skin aging, including epinephrine bitartrate, clotrimazole, dacarbazine, prilocaine, prilocaine hydrochloride, becaplermin, cisplatin, eflornithine hydrochloride and etoposide phosphate (Figure 5C).

## 4 Discussion

We observed differential expression of MT family genes (including MT1A, MT1G, MT1E, MT1X) in the basal cell C11 cohort, alongside significant upregulation of zinc and copper responses in the aged group within this cohort. MTs [9] are low molecular weight proteins with antiapoptotic properties that scavenge free radicals. Their functions extend beyond metal ion homeostasis to include the promotion of cell survival, tissue regeneration, metabolic activity, and the attenuation of inflammatory and apoptotic pathways [10, 11]. A single‐cell RNA‐seq study of the thyroid gland revealed a positive correlation between the expression of MT genes and age, attributed to the senescence of thyroid epithelial cells. These genes are essential for maintaining thyroid homeostasis during aging, and their increased expression protects thyrocytes from iodine stress in vitro [12]. Although MT1 is known to protect against UV damage [13], its uniform protein distribution implies broader homeostatic roles beyond photoprotection in aged skin. Our IHC results showed uniform MT1 protein distribution despite subcluster‐specific mRNA patterns in scRNA‐seq. This suggests potential posttranslational regulation or compensatory mechanisms among MT isoforms. Further validation with spatial transcriptomics or isoform‐specific antibodies is needed.

Furthermore, we identified increased regulatory activity of the FOSB(+) and DDIT3(+) regulons in basal cell cluster C1. The gene regulatory network analysis showed that MT genes were targets of these regulons, linking FOSB/DDIT3 activation to metal ion homeostasis in aging keratinocytes. The consistent upregulation of FOSB at both transcriptional (scRNA‐seq) and protein (IHC) levels establishes it as a keratinocyte‐specific aging biomarker, distinct from fibroblast‐centric mechanisms reported previously [4, 5]. Given the concurrent dysregulation of MT isoforms (e.g., basal C11‐specific mRNA patterns) and their functional roles in oxidative stress response, our findings suggest a coordinated FOSB‐MT axis may drive keratinocyte aging through metal ion‐dependent pathways.

Drugged‐cell response prediction was performed to identify new therapeutic targets. Metformin exerts therapeutic benefits against skin aging by improving insulin sensitivity, promoting cellular repair, and modulating anti‐inflammatory pathways [14, 15]. Drug–response analysis revealed heightened activity in secretory (C3) and vellus hair follicle cells (C12), with aged basal keratinocytes (C6) and melanocytes (C18) demonstrating particularly robust responsiveness. These findings highlight metformin's multi‐mechanistic potential in tackling skin aging. Notably, the cell‐type‐specific enhancement of drug activity provides critical insights into population‐dependent therapeutic effects, fostering its translation into targeted antiaging therapeutic strategies. Diclofenac [16, 17] preferentially targeted basal keratinocytes in our analysis, consistent with its multi‐target capacity to modulate matrix metalloproteinases (MMPs) including the aging‐associated MMP7. The basal cell‐specific activity aligns with diclofenac's clinical efficacy in improving photodamaged skin, suggesting targeted therapeutic potential for age‐related epidermal changes.

Through integrated single‐cell transcriptomics and computational drug prediction, we also revealed therapeutic strategies against skin aging. Epinephrine bitartrate emerged as a promising repurposing candidate, demonstrating targeted activity in basal and spinous keratinocytes. This aligns with the endogenous role of epinephrine in keratinocytes, where it modulates cAMP signaling to regulate epidermal proliferation, differentiation, and stress responses [18]. The predicted activation of cAMP‐mediated pathways suggests its potential to counteract age‐related dysregulation of keratinocyte homeostasis, particularly in FOSB(+) cell populations exhibiting stress‐associated signatures. Notably, epinephrine's dual capacity to influence both developmental [19] and stress‐adaptive processes [20] positions it as a multifaceted intervention for aging skin, where compromised barrier function and chronic stress signaling converge. These findings highlight a targeted strategy to restore epidermal integrity while mitigating stress‐induced senescence. The second prioritized agent, becaplermin, showed specific activity in dermal fibroblasts (C16) and basal cells, supporting its potential for combating age‐related extracellular matrix deterioration while leveraging its established wound‐healing efficacy [21]. Retinol emerged as another compelling option due to its spinous cell‐specific effects on differentiation markers (KRT10/SPINK5), complementing epinephrine's barrier repair potential.

While our study provides a comprehensive single‐cell transcriptomic atlas of human skin aging, several limitations should be noted. First, the sample size, though sufficient for initial discovery, may not fully capture the heterogeneity of aging across diverse populations. Second, although we have confirmed the increased regulatory activity of FOSB(+) regulons in aged basal keratinocytes and its association with MT family genes, the causal relationship of FOSB in skin aging remains unverified, which limits the depth of our understanding of its molecular mechanism. Future studies incorporating spatial transcriptomics, proteomics, and functional genetic experiments would help validate and extend these findings.

Our integrated single‐cell and IHC approach identified FOSB as a validated keratinocyte aging marker, while revealing unexpected discordance between MT1 mRNA and protein patterns. The drug‐target pipeline prioritizes compounds like epinephrine bitartrate for future validation, with particular emphasis on their effects on FOSB(+) keratinocyte subpopulations.

## Author Contributions

Y.Z. designed and performed experiments, and wrote the paper. Y.W. provided the methodology and provided technical and material support. D.L. analyzed the data. Y.G. and R.Z. conducted the data collection and analysis, designed and organized the study and checked the manuscript. All authors have read and approved the final manuscript.

## Funding

The work was supported by grants from the National Natural Science Foundation of China (No. 82201993).

## Conflicts of Interest

The authors declare no conflicts of interest.

## Supporting information


**Figure S1:** UMAP plot of subpopulations, and scaled gene expression levels in each subcluster where red color indicates high expression levels.

**Figure S2:** Immunohistochemistry (IHC) staining of MT1 in young and elder skin.

**Figure S3:** Dot plot showing the scaled expression of representative cell type markers for each population.

**Figure S4:** Mirror bar plot showing the enriched GO terms using the top 30 DEGs from each cell type.

**Figure S5:** Regulon specificity score (RSS).

**Figure S6:** Histogram indicating different regulons (transcription factor and the target genes) changes by comparing elder cells (red) and young cells (blue), where the y‐axis showed the AUC regulons activity and the x‐axis showed the density. Names colored in blue represented the regulons showed decreased activity in aged cells whereas names in red showed the regulons had increased TF activity in aged samples.

**Figure S7:** Gene expression changes between the elder group and the young group, in which the y‐axis showed a subset of composited genes in the regulons.

**Figure S8:** IHC staining of LTF in young and elder skin.

**Figure S9:** IHC staining of FOSB in young and elder skin.

**Figure S10:** IHC staining of JAG1 in young and elder skin.

## Data Availability

The data that support the findings of this study are available from the corresponding author upon reasonable request.
