# Supplementary figures and images for: Single‐Cell Atlas of Aging Human Skin Reveals FOSB‐Related Transcriptional Programs and Druggable Targets
Source: J Cosmet Dermatol. 2025 Nov 26;24(12):e70569. doi: 10.1111/jocd.70569 (PMC12648372; doi:10.1111/jocd.70569)

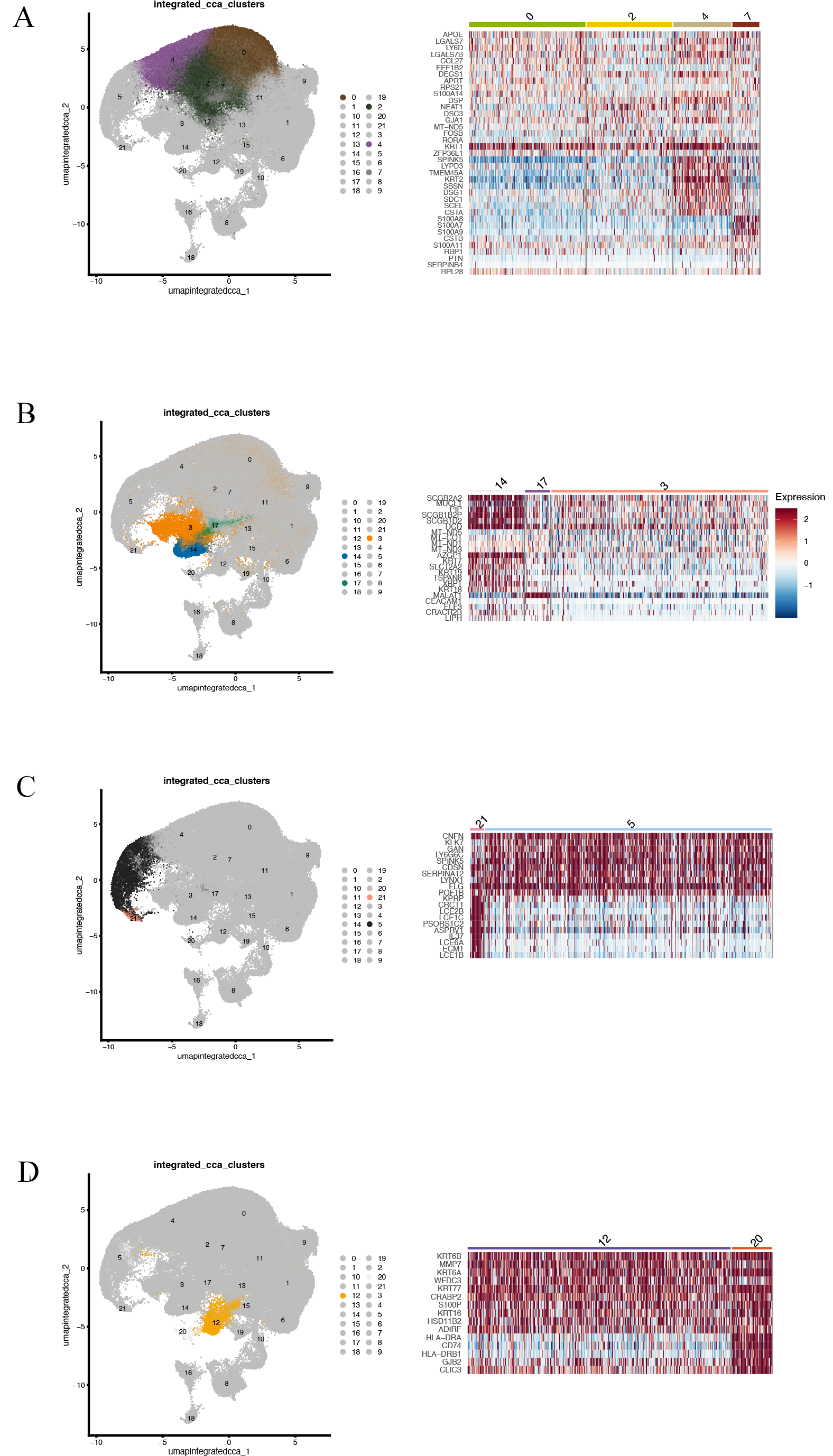

Supplement: Supplementary file 1 — Figure S1: UMAP plot of subpopulations, and scaled gene expression levels in each subcluster where red color indicates high expression levels. [file JOCD-24-e70569-s008.tif]

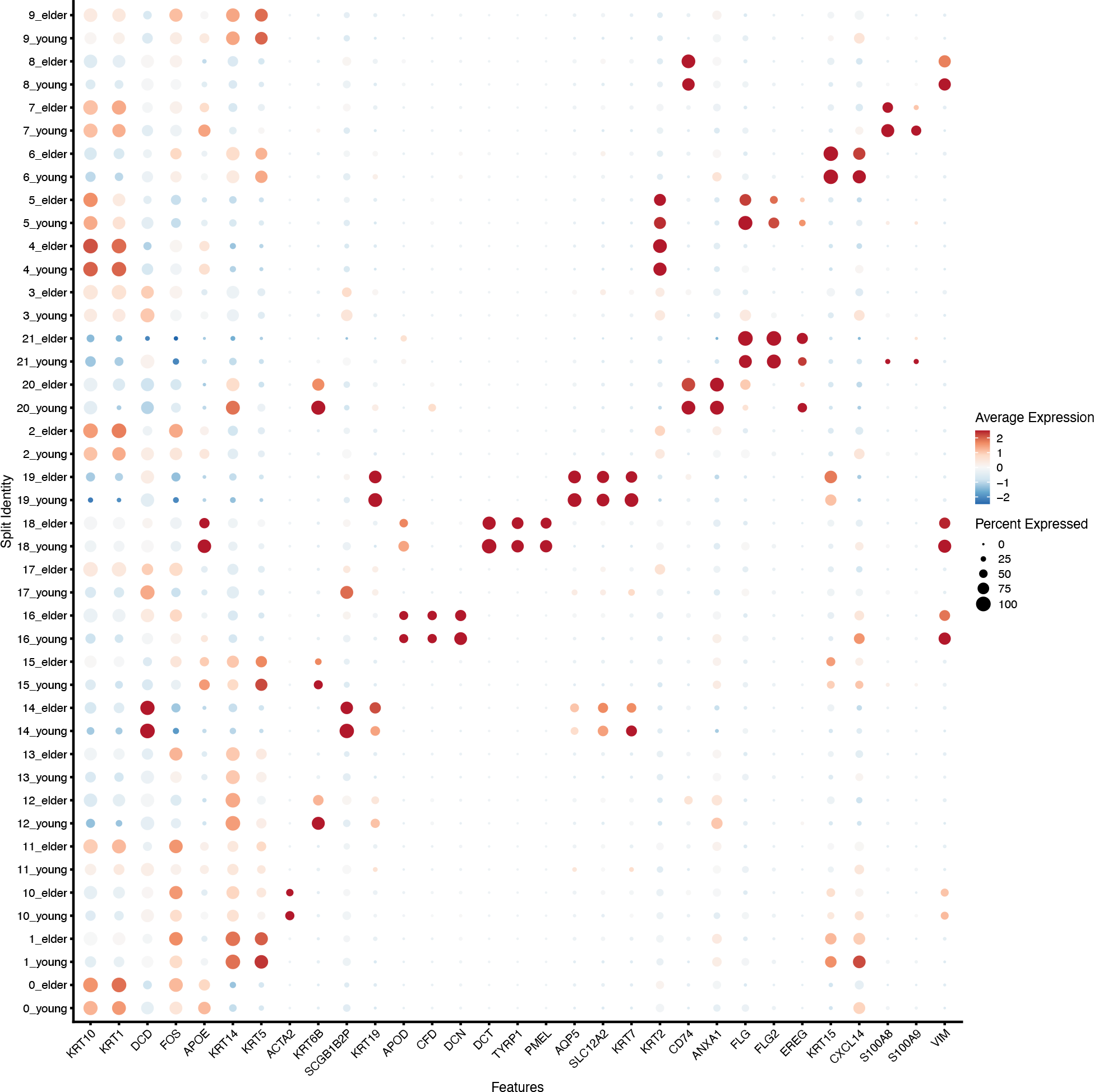

Supplement: Supplementary file 3 — Figure S3: Dot plot showing the scaled expression of representative cell type markers for each population. [file JOCD-24-e70569-s003.tif]

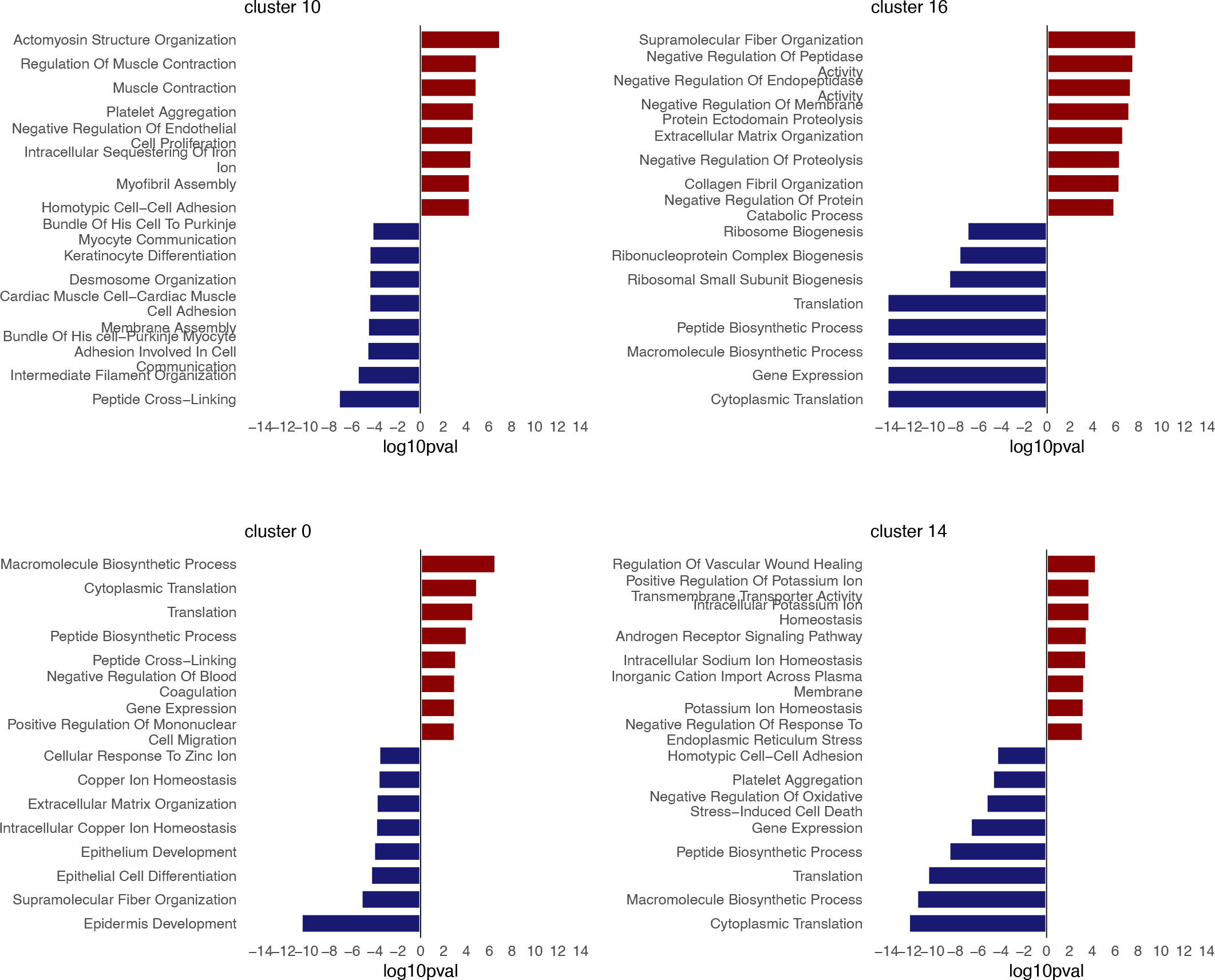

Supplement: Supplementary file 4 — Figure S4: Mirror bar plot showing the enriched GO terms using the top 30 DEGs from each cell type. [file JOCD-24-e70569-s010.tif]

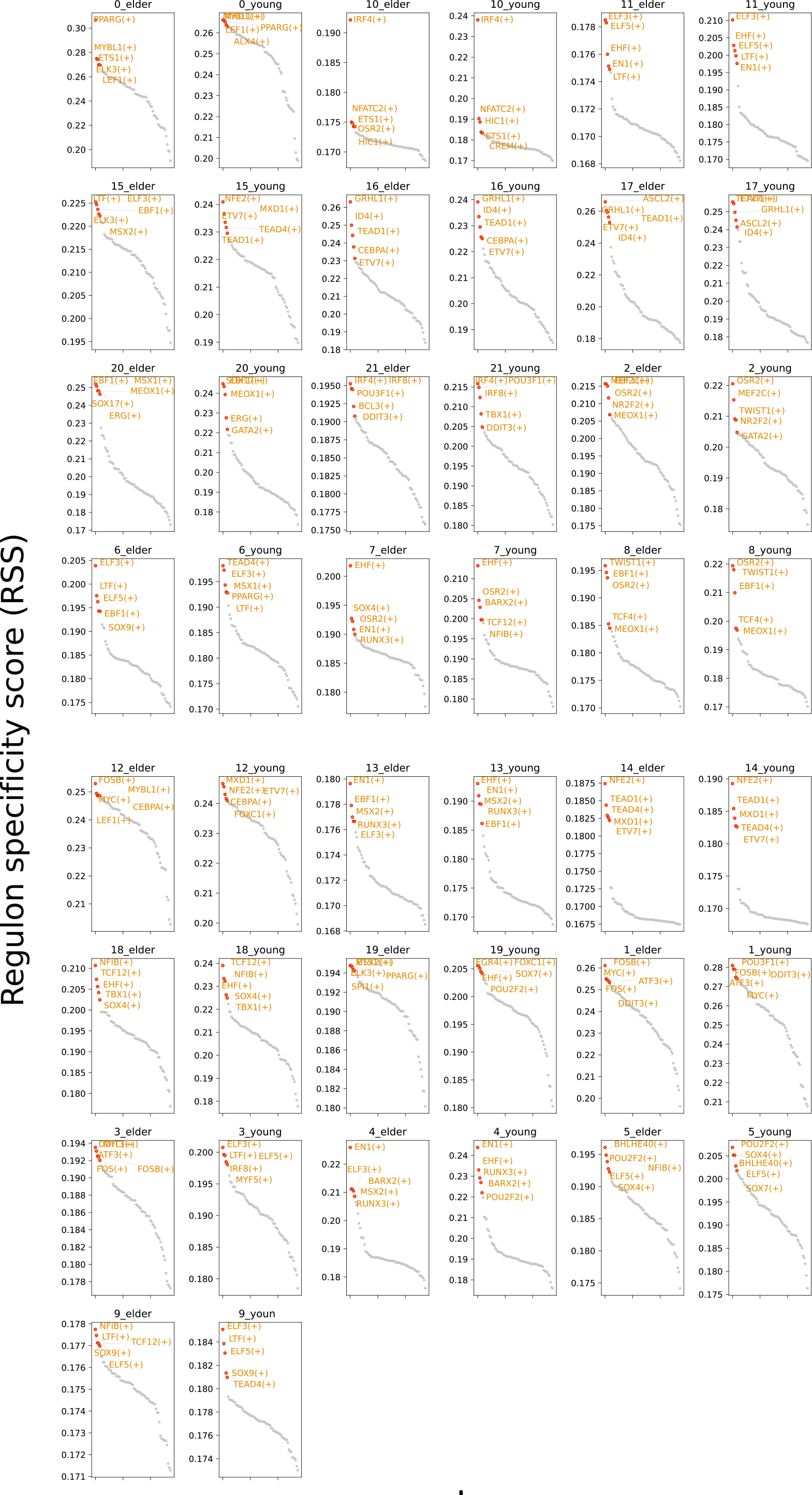

Supplement: Supplementary file 5 — Figure S5: Regulon specificity score (RSS). [file JOCD-24-e70569-s001.tif]

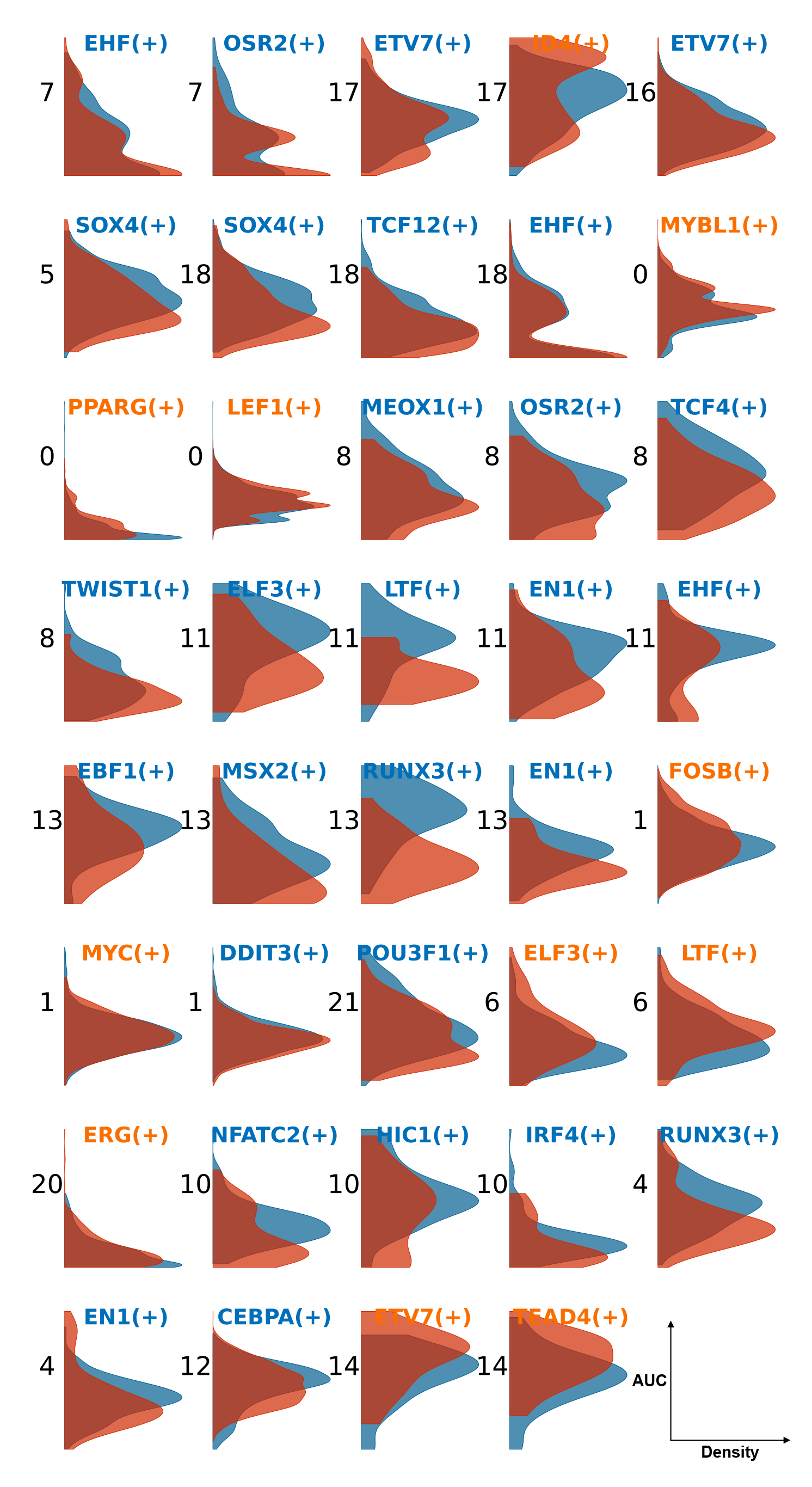

Supplement: Supplementary file 6 — Figure S6: Histogram indicating different regulons (transcription factor and the target genes) changes by comparing elder cells (red) and young cells (blue), where the y‐axis showed the AUC regulons activity and the x‐axis showed the density. Names colored in blue represented the regulons showed decreased activity in aged cells whereas names in red showed the regulons had increased TF activity in aged samples. [file JOCD-24-e70569-s009.tif]

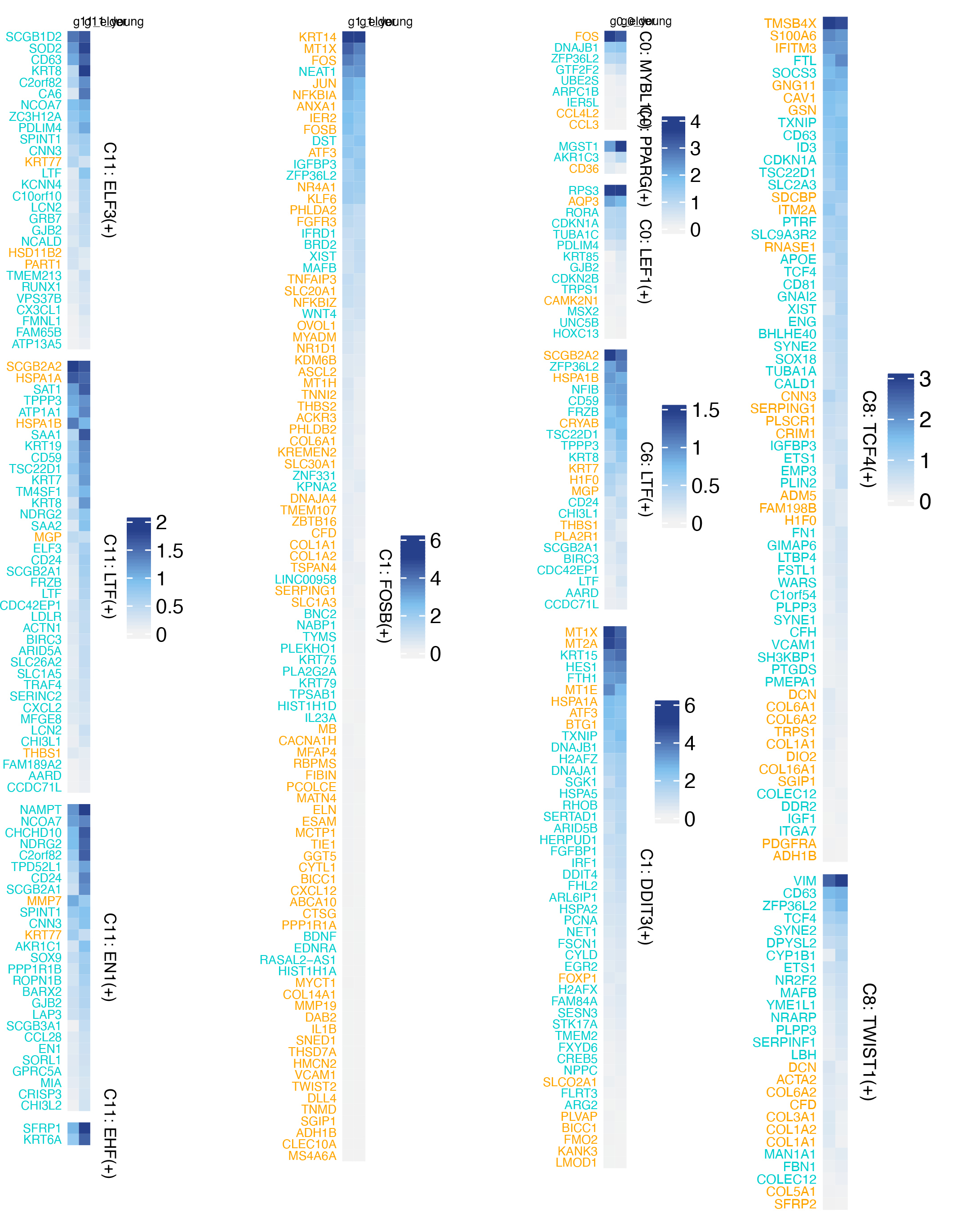

Supplement: Supplementary file 7 — Figure S7: Gene expression changes between the elder group and the young group, in which the y‐axis showed a subset of composited genes in the regulons. [file JOCD-24-e70569-s004.tif]
